# Supplementary material for: Normalizing Untargeted Periconceptional Urinary Metabolomics Data: A Comparison of Approaches
Source: Metabolites. 2019 Sep 21;9(10):198. doi: 10.3390/metabo9100198 (PMC6835889; doi:10.3390/metabo9100198)
Supplement: Supplementary file 1 [file metabolites-09-00198-s001.pdf]

metabolites

MDPI

Article

Normalizing Untargeted Periconceptional Urinary Metabolomics Data: A Comparison of Approaches

Ana K. Rosen Vollmar [1], Nicholas J. W. Rattray [1,2], Yuping Cai [1], Álvaro J. Santos-Neto [1,3], Nicole C. Deziel [1], Anne Marie Z. Jukic [4] and Caroline H. Johnson [1,*]

[1] Department of Environmental Health Sciences, Yale School of Public Health, New Haven, CT 06510, USA; ana.rosenvollmar@yale.edu (A.K.R.V.); nicholas.rattray@strath.ac.uk (N.J.W.R.); ping.cai@yale.edu (Y.C.); alvarojsn@iqsc.usp.br (A.J.S.N.); nicole.deziel@yale.edu (N.C.D.)

[2] Strathclyde Institute of Pharmacy and Biomedical Sciences, University of Strathclyde, G4 0RE Glasgow, UK

[3] São Carlos Institute of Chemistry, University of São Paulo, São Carlos, SP, 13566-590, Brazil

[4] Epidemiology Branch, National Institute of Environmental Health Sciences, Durham, NC 27709, USA; jukica@niehs.nih.gov

* Correspondence: caroline.johnson@yale.edu

Supplementary Materials

Figure S1. Results of 200 random permutation tests (permutation by implantation status) performed on RPLC datasets for each normalization method.

Figure S2. Results of 200 random permutation tests (permutation by implantation status) performed on HILIC datasets for each normalization method.

Table S1. Difference in the original R^2 from OPLS-DA, and the R^2 calculated from the permutation test.

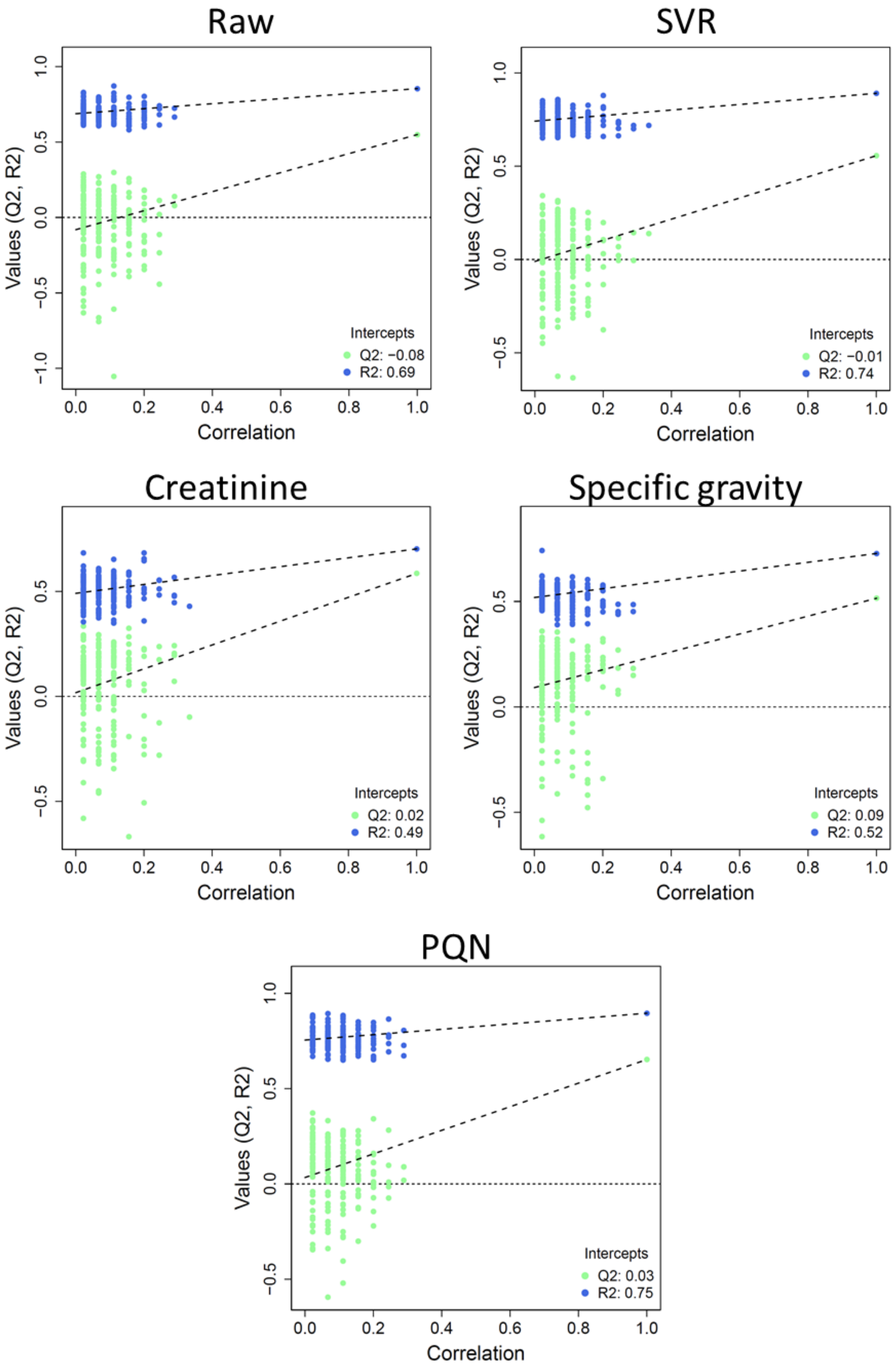

Figure S1. Results of 200 random permutation tests (permutation by implantation status) performed on RPLC datasets for each normalization method. The vertical axis corresponds to the Q^2 and R^2 values from OPLS-DA analysis, with original values in the upper right-hand corner. The horizontal axis is the correlation coefficient between the original and permuted values. RPLC, reversed-phase liquid chromatography; OPLS-DA, orthogonal partial least-squares discriminant analysis.

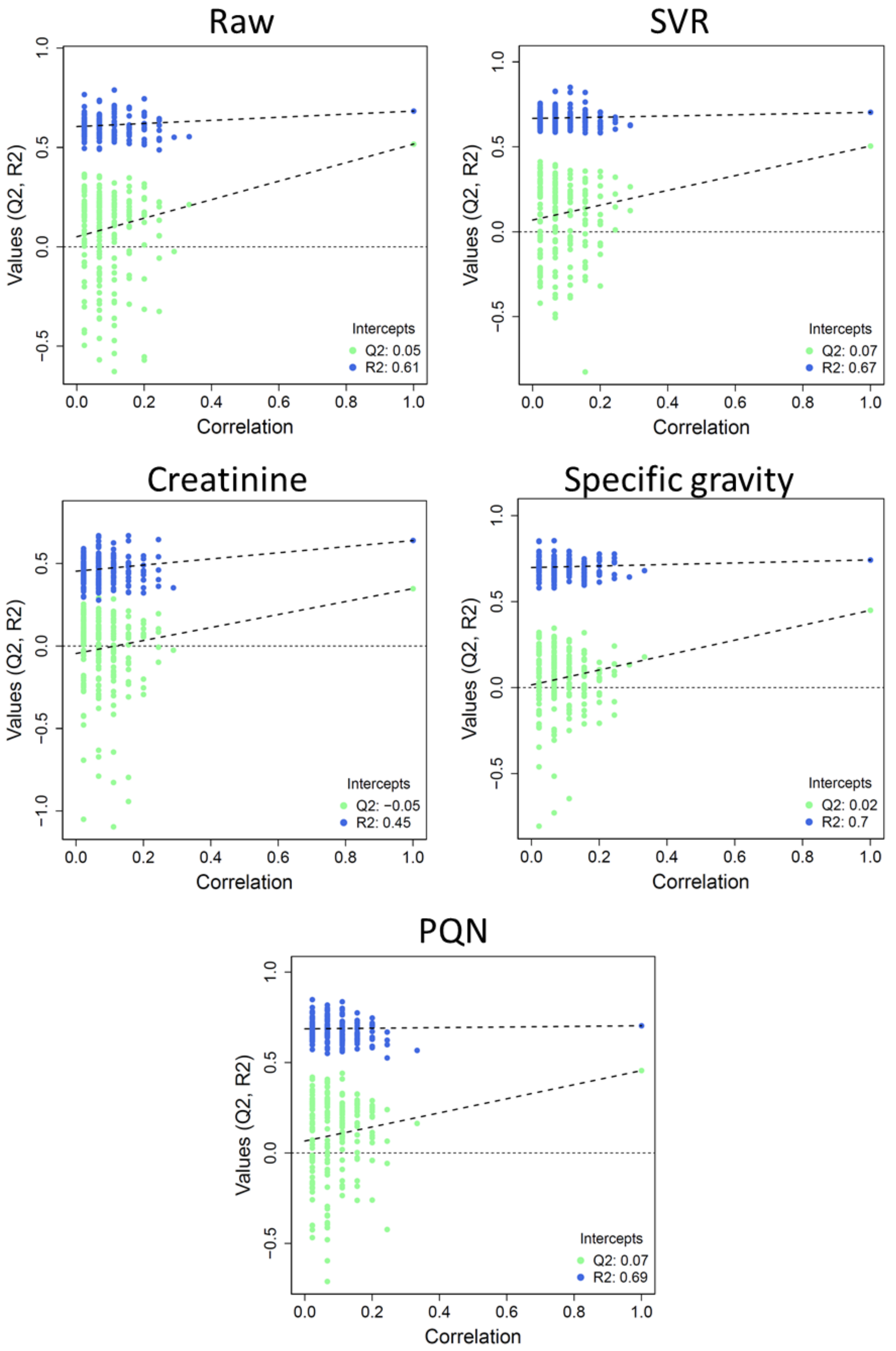

Figure S2. Results of 200 random permutation tests (permutation by implantation status) performed on HILIC datasets for each normalization method. The vertical axis corresponds to the Q^2 and R^2 values from OPLS-DA analysis, with original values in the upper right-hand corner. The horizontal axis is the correlation coefficient between the original and permuted values. HILIC, hydrophilic interaction chromatography; OPLS-DA, orthogonal partial least-squares discriminant analysis.

Table S1. Difference in the original R^2 from OPLS-DA, and the R^2 calculated from the permutation test.[1]

Normalization approach	RPLC data			HILIC data		
	Original R^2	Permuted R^2	Difference	Original R^2	Permuted R^2	Difference
Raw	0.93	0.69	0.24	0.87	0.61	0.26
SVR	0.95	0.74	0.21	0.90	0.67	0.23
Creatinine	0.82	0.49	0.33	0.75	0.45	0.30
Specific gravity	0.86	0.52	0.34	0.87	0.70	0.17
PQN	0.94	0.75	0.19	0.91	0.69	0.22

[1] Abbreviations: OPLS-DA, orthogonal partial least-squares analysis; RPLC, reversed-phase liquid chromatography; HILIC, hydrophilic interaction chromatography; SVR, support vector regression; PQN, probabilistic quotient normalization. Raw data were processed using XCMS. SVR normalization was then applied to all datasets. Creatinine, specific gravity, and PQN adjustments were carried out after SVR normalization.

© 2019 by the authors. Submitted for possible open access publication under the terms and conditions of the Creative Commons Attribution (CC BY) license (http://creativecommons.org/licenses/by/4.0/).
